# Evaluation of the Drug Treatment and Persistence of Onychomycosis

**DOI:** 10.1100/tsw.2004.134

**Published:** 2004-08-31

**Authors:** Andrew W. Campbell, Ebere C. Anyanwu, Mohammed Morad

**Affiliations:** ^1^ Medical Center for Immune and Toxic Disorders, 20510 Oakhurst Drive, Suite 200, Spring TX, USA; ^2^ Neurosciences Research, Cahers Inc., 8787 Shenandoah Park Drive, Suite 122, Conroe, Houston, 77385 TX, USA; ^3^ Clalit Health Services and Division of Community Health, Department of Family Medicine, Ben Gurion University, Beer-Sheva, Israel

**Keywords:** onychomycosis, persistence, toxigenic molds, antifungal drugs, resistance

## Abstract

Onychomycosis is a common nail disease responsible for approximately 50% of diseases of the nail. It occurs more in the elderly, though several cases have been reported among children. Several factors influence, such as climate, geography, and migration. The two dermatophytes most commonly implicated in onychomycosis are Trichophyton rubrum and T. mentagrophytes, accounting for more than 90% of onychomycoses. Nonetheless, several other toxigenic molds have been implicated. For convenience, onychomycosis is divided into four major clinical presentations: distal subungal, which is the most common form of the disease; proximal subungal, which is the most common form found in patients with human immunodeficiency virus infection; superficial; and total dystrophic onychomycosis. Epidemiology of onychomycosis in adults and children is evaluated and the most common clinical symptoms addressed. Although the risk factors are discussed, the multifactorial nature of onychomycosis makes this inexhaustible. The diagnosis and treatments are difficult and the choice of appropriate antifungal drugs complex and require the knowledge of the chemical structures of the metabolites of the molds that cause onychomycosis and their interaction with the antifungal drugs. This is true because most of the antifungal drugs are derived from mold/fungal metabolism. Treatment with griseofulvin and amphotericin is displaced by the use of newer drugs from azole compounds, pyrimidines, and allylamines derivatives. Amorolfine, itraconazole, and ciclopirox nail lacquer solution 8 have gained support globally, but the side effects, drug resistance, and persistence of the disease are still a serious concern to the patients, just as economics and quality of life. Hence, the search for safer and more efficacious drug treatments are continuing.

## INTRODUCTION

Onychomycosis is a fungal infection that can lead to thickening, splitting, roughening, and discoloration of the nail. The causative toxigenic molds are mainly the dermatophytes, saprophytes, and yeasts. The dermatophytes that can cause onychomycosis (*Trichophyton*, *Epidermophyton,* and *Microsporum* genera) have the ability to colonize keratinized tissues of the human body, such as skin, hair, and nails. These toxigenic molds produce keratinases that allows them to break down keratin in the stratum corneum for penetration. The host immune system can affect dermatophyte pathogenicity and when there are defects in the immune system, locally invasive dermatophyte diseases may ensue. Of the dermatophytes, *T. rubrum* and *T. mentagrophytes* are the most frequently reported causes of onychomycosis, which accounts for a reported 90% of toenail infections[1,2,3]. In 78% of culture-positive cases, a dermatophyte was isolated as the causative agent, in 10% yeasts, and in 7% molds[4]. The term "tinea unguium" is used specifically to describe invasive dermatophytic onychomycosis.

The most common clinical presentations are distal and lateral subungual onychomycosis, which usually affects the great/first toe and white superficial onychomycosis, which generally involves the third/fourth toes[3,5]. When the infection is due to a dermatophyte, both "ringworm of the nail" and "tinea unguium" are sometimes used as synonyms. The most common clinical symptoms of onychomycosis, both in mycologically proven and nonproven cases, were discolorization of nail plate, hyperkeratosis, and brittle nails. The number of infected toenails positively correlated with the age of the patient. The high occurrence of mixed infections, clinical symptoms characteristic to long-lasting or chronic infection, and high number of infected toenails indicate that the patients have more advanced stages of toenail onychomycosis compared with other western and central European countries[4]. Onychomycosis is very persistent and often resistant to antifungal drugs. The reasons for this persistence are often underestimated, but difficult to identify.

## AIMS OF THIS REVIEW

The aims of this paper were to review the epidemiology of onychomycosis, to understand the extent to which the disease is distributed, to evaluate the disease management and drug treatment, and to suggest possible reasons for the disease persistence. The risk factors will be assessed to preclude our understanding of the pharmacoeconomic impact of the disease on humans. Efforts will also be made to address the impact of the disease on health-related quality of life.

## EPIDEMIOLOGY OF ONYCHOMYCOSIS

The overall incidence in the population is not definite, but it is reported at about 2–13% and presently on the rise[6,7]. The incidence of fungal nail infections is increasing and this is possibly because of several factors: better methods of detection, a growing population of immunocompromised patients who have a greater susceptibility to such infections, the increased use of immunosuppressive drugs, the increasing number of elderly people, worldwide travel, and the use of communal bathing facilities[2]. The prevalence of onychomycosis in the world's population is reported from 2–18% or higher and accounts for approximately 50% of all nail disorders[8] and may affect up to 30% of the population by age 60 years[1].

Although nonfungal diseases such as repeated trauma, psoriasis, lichen planus, local tumors, vascular disorders, and inflammatory diseases can manifest similar symptoms as found in onychomycosis, about 50% of all nail diseases seen in practice are caused by onychomycosis and more than 99% of these cases caused by dermatophytes (most common are *T. rubrum* and *T. mentagrophytes*)[1]. In some geographical areas, it is reported that in more than 80% of nail disease cases, onychomycosis is caused by these dermatophytes[8]. The toenails are affected approximately four times as often as those of the fingernails[9,10]. Onychomycosis is common in the U.S. and although dermatophytes remain the most common cause, *T. rubrum* is apparently the most prevalent fungal pathogen[11]. Recent surveys done on a large scale in Europe in adults indicated a high prevalence of 20–30% and frequently observed more in adults, especially among the elderly[12]. In western Europe and as well as in North America, onychomycosis involves principally the feet, especially in males. By contrast, in southern Europe, in the Middle and Far East, the prevalence is highest in women's fingernails and is often associated with paronychia[12].

Overall, in Europe, the prevalence of onychomycosis is on the increase yearly as shown in a large-scale, time-determined study by Burzykowski et al.[13]. They found that in the study I, 57.0% of patients had at least one foot disease. In study II, 61.3% had at least one foot disease. The proportions of patients with fungal foot disease and nonfungal foot disease in study I was 34.9 and 38.4%, respectively, and in study II 40.6 and 41.7%, respectively. Orthopedic conditions and metatarsal corns were the most frequently reported, and onychomycosis and tinea pedis were the most frequently observed fungal infections[13]. The prevalence of these conditions was 20%, respectively, and it increased with advancing age with more men than women having fungal infection of the feet[14]. Genetics has also been identified as a factor governing the epidemiology of onychomycosis; *T. rubrum* infection has shown a familial pattern of autosomal dominant inheritance[5]. Disease and lifestyle may also play a role in the epidemiology of fungal nail infections. Studies have shown that diabetes, acquired immunodeficiency syndrome, and peripheral arterial disease may be independent predictors of onychomycosis. Because of the multifactorial nature of the epidemiology, accurate diagnosis, pertinent treatment, and patient education must be paramount when treating the disease[5]. In India, the prevalence of onychomycosis in the diabetic and control groups was 17 and 6.8%, respectively, the difference being statistically significant ($p < 0.001$). The presence of onychomycosis was found to correlate significantly with increasing age ($p < 0.01$) and male gender ($p < 0.05$) in both diabetic and control groups. From diabetics, yeasts were the most common isolate (48.1%), followed by dermatophytes and nondermatophyte molds in 37 and 14.8%, respectively[15]. Diabetics were also found to be 2.5 times more likely to have onychomycosis than the controls[15,16,17,18].

## PEDIATRIC EPIDEMIOLOGY

There are still little data available about the epidemiology of childhood onychomycosis. However, out of a sample of 963 children, 296 had proven onychomycosis and more than three-fourths of these were found in children more than 6 years old with boys more frequently affected than girls[19]. *T. rubrum* was also the main pathogen, followed by *Candida* spp. and *T. interdigitale*. As in adults, onychomycosis is probably the main nail disease in children. After the age of 6 years, the presentation is very similar to that in adults[19]. The prevalence of onychomycosis in a sample of North American children 18 years old or younger was 0.44% (n = 2,500)[20]. Twenty-six children with onychomycosis were reported[21] of which 16 were boys (61.4%) and 10 were girls (38.6%). The most frequent variety was distal subungual onychomycosis (88.5%) and the earliest sign was distal subungual hyperkeratosis. In children, the agent most frequently isolated is the *T. rubrum*, which was found in 18 cases (69.2%)[22,23].

In Japan and France, the clinical features of onychomycosis vary with the age of the child and after the age of 12 years, onychomycosis become more common[24,25].

## RISK FACTOR IN ONYCHOMYCOSIS

The prevalent predisposing risk factors are sports participation, average temperature, and family history of tinea pedis. The predisposing diseases are hypercholesterolemia, cardiovascular disease, diabetes mellitus, and osteoarticular disease. The risk factors associated with a poor response to antifungal therapy may be subdivided into (a) genetic, (b) environmental, (c) systemic conditions, (d) local nail characteristics, and (e) other miscellaneous items[26].

**TABLE 1**
**The General Risk Factors for Any Type of Onychomycosis**

- Increasing age
- Male gender
- Diabetes
- Nail trauma (onychogryphosis)
- Hyperhydrosis
- Peripheral vascular diseases
- Poor hygiene
- Tinea pedis especially the "moccasin type"
- Immunodeficiencies
- In the case of candidal onychomycosis in particular, chronic exposure of the nails to water can be a significant risk factor

**TABLE 2**
**Summary of the Reported Characteristics of Onychomycosis [1,27,28]**

| Type | Distal Subungual Onychomycosis (DSO) | Proximal Subungual Onychomycosis (PSO) | White Superficial Onychomycosis (WSO) |
|---|---|---|---|
| Frequency | It is the most common clinical form. | Uncommon in the general population but very frequent in AIDS patients. | Represents about 10% of cases of onychomycosis. |
| Evolution | Infection begins with invasion of the hyponychium (the place where the nail separates from the nail bed). | Infection by invasion of the proximal nail fold, with subsequent penetration into the newly forming nail plate that is underneath. The distal nail remains normal until late in the disease. | Infection begins at the superficial layer of the nail plate invading progressively deeper layers. |
| Clinical appearance | Onycholysis (separation of the nail plate from the nail bed) and thickening of subungueal area; when superinfection with bacteria and/or molds occurs, the nail plate turns yellowish brown. | Subungual hyperkeratosis, leukonychia, proximal onycholysis, and destruction of the nail unit. | Initially "white islands" are seen on the external nail plates. These gradually coalesce until the entire nail plate is involved. |
| Most common etiologic agent | *T. rubrum* | *T. rubrum* | *T. mentagrophytes, Aspergillus terreus, Acremonium roseogriseum, Fusarium oxysporum* |
| Affected nails | Toenails are most commonly affected, but may affect fingernails as well. | Much more common on the toenails; rarely affects fingernails. | Mainly affects toenails. |

Among the factors promoting fungal nail invasion, some are local depending on the patient (trophic troubles or circulatory impairment overlapping of digits), some are due to general factors such as immunosuppression, diabetes mellitus, or psoriasis. There are also behavioral factors (occupation, lifestyle, sports), which favor the meeting with the pathogenic fungus[12]. Several other risk factors are associated with onychomycosis. A history of the following factors has more than doubled the risk of onychomycosis, namely, cancer (OR 3.44; 95% CI 1.15–10.35), psoriasis (OR 2.44; 95% CI 1.61–3.72), tinea pedis interdigitalis (OR 3.93; 95% CI 3.11–4.95), the moccasin form of tinea pedis (OR 4.26; 94% CI 3.34–5.45), parents with onychomycosis (OR 2.59; 95% CI 1.89–3.53), children with onychomycosis (OR 3.48; 95% CI 2.05–5.88), spouse with onychomycosis (OR 2.53; 95% CI 1.72–3.72), regular swimming activity (OR 2.57; 95% CI 2.00–3.30), and age 50 years or older (OR 2.74; 95% CI 2.19–3.42)[17].

## DIAGNOSIS

Onychomycosis continues to be a difficult diagnosis to establish and treat. However, a definitive diagnosis is crucial for effective treatment because many other skin and nail disorders mimic onychomycosis. Diagnosis involves microscopic potassium hydroxide preparation, cultures, nail biopsy, and histologic analysis[1,29]. Hence, microscopy and culture are regarded as the diagnostic “gold standards” for onychomycosis, although biopsy of the nail may be required to obtain a definitive diagnosis when conditions that mimic onychomycosis, such as psoriasis, are suspected[27].

Using this diagnostic method, four types of onychomycosis are recognized based on the site and pattern of fungal invasion. Dermatophyte fungi are the predominant pathogens, but yeasts (especially *C. albicans*) and nondermatophyte molds may also be implicated. Accurate diagnosis requires direct microscopy and fungal culture. The differential diagnosis includes psoriasis, lichen planus, onychogryphosis, and nail trauma[28].

## TREATMENT

Onychomycosis is difficult to treat and the search for new treatment modalities and drugs is hampered by the lack of understanding of the basic pathophysiological mechanisms that underlie these frequently encountered infections. In some patients, the infections are more persistent due to a number of interacting factors: patient susceptibility, fungal growth patterns that resist treatment, and the occurrence of dormant fungal spores (arthrospores) in the nail[30,31]. Although new antimycotic agents are far more promising than the ones used in earlier treatments, relapse rates still remain high[32]. Treatment can include topical and systemic antifungal therapies as well as nonpharmaceutical methods[29].

A number of modern treatment strategies are available and are generally well tolerated and effective. However, a significant proportion of patients (20–30%) can expect treatment failure and/or relapse following treatment[30]. Therefore, the treatment approach needs to take into account the location and extent of onychomycosis, sensitivity of drug to fungal organism, adverse-effects profile, dosage schedule, duration of therapy, concomitant medical conditions, and concurrent medications[33]. Nevertheless, onychomycosis has been treated for years with oral antifungal agents and more recently in the U.S. with a topical nail lacquer. However, until recently, available therapies were inadequate due to low cure rates, high relapse rates, and often-dangerous side effects. An increased understanding of nail pharmacokinetics has led to the development of safer, more effective systemic therapies for onychomycosis, such as itraconazole, fluconazole, and terbinafine.

These new oral antifungal agents allow shorter periods of treatment, provide rapid efficacy, and may improve patient compliance and attitudes regarding therapy. However, complete treatment will depend on several factors, including appropriate spectrum of activity, adverse effects, and potential drug interactions plus patient preferences for specific dosing regimens[34,35,36].

## Antifungal Drugs and Some Side Effects: Structural and Functional Groups Consideration

The structural and functional properties of mycotoxins produced by toxigenic molds and their interactive health implications with antifungal drugs have been reported[37]. In that report, we showed that it was most likely that the interactions of mycotoxins with antifungal drugs may, at least in part, contribute to the observable persistent illnesses, antifungal drug resistance, and allergic reactions in patients exposed to chronic toxigenic molds. Most of the available antifungal drugs are derived from molds and these have structural and functional group similarities to those of mycotoxins. Therefore, to increase our understanding of these facts, the drugs are briefly introduced and assessed below. Perhaps the knowledge of their interactions could be necessary in choosing the appropriate antifungal drugs for the patients, enhancing the accurate treatment of the disease and prevention of unnecessary side effects[37]. Thus, the antifungal drugs are classified under the following chemical functional groups.

### *Polyene Compounds*

The examples are amphotericin B (Fig. 1) and nystatin. Both of these drugs are similar in structure, but slightly dissimilar in their functional group. Amphotericin B (fungizone) binds to ergosterol moiety in the plasma membrane causing derangement of the membrane integrity and leakage of cytoplasmic contents. Administered systemically.

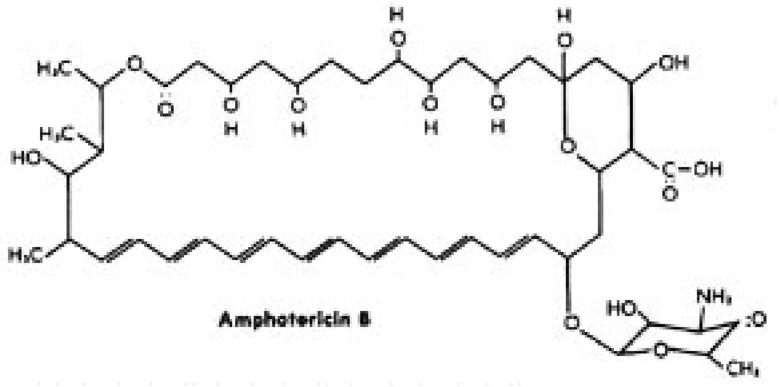

**FIGURE 1.** Amphotericin B.

Even though both of these drugs are said to be effective against most fungal infections including candidad and dermatophytes (*Epidermophyton, Trichophyton, Microsporium*), they have various side effects including:

- Hypersensitivity-anaphylaxis-hematological
- Fever, chills, headache, nausea
- Thrombophlebitis
- Hemolytic anemia
- Renal toxicity
- Hepatic damage
- Hypokalemia

## Griseofulvin

Griseofulvin (Fig. 2) was the first significant oral agent available to manage onychomycosis in the U.S. It reacts by binding to the microtubules comprising the spindles and inhibits mitosis. It also incorporates into the affected keratin, hence it is described as fungistatic. However, it is not as efficacious as the newly developed drugs and it must be given for 1 year or more to be effective. Even when used long term, it is effective in only about 30% of patients[38]. Low cure rates are related to poor bioavailability and the fungistatic rather than fungicidal effect of the drug.

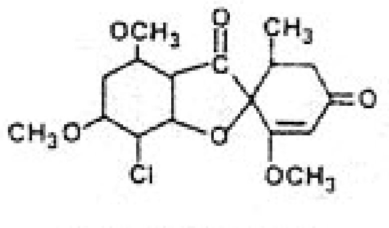

**FIGURE 2.**

Topical treatment of onychomycosis allows the patient to apply medication directly to the affected area, thereby decreasing the potential for adverse events and drug interactions. Historically, several topical antifungal agents have been used in the treatment of onychomycosis, but the evidence for their effectiveness is based on very limited data or anecdotal reports. Recently, the development of new, effective topical agents has renewed interest in this form of therapy[39,40,41].

## Amorolfine

Amorolfine is a new topical antifungal of the phenylpropyl morpholine class, which is highly active both *in vitro* and *in vivo* against yeasts, dermatophytes, and molds responsible for superficial fungal infections[42]. Amorolfine has a persistent antifungal effect in the nail bed and in the skin without being systemically absorbed[42]. This has been confirmed by clinical work showing that amorolfine is effective in treating dermatomycoses and onychomycoses, when administered as cream or nail lacquer. A 5% concentration of amorolfine nail lacquer was found to produce a better cure rate in onychomycosis than a lower concentration of 2%[29,42]. Amorolfine has 5% nail lacquer formulation and the most significant advantage of nail lacquer formulation is that it builds a nonwater-soluble film on the nail plate, which remains at the application site for a week. This film acts as a depot for the drug[43].

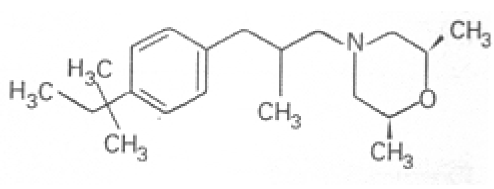

**FIGURE 3.** The chemical structure of amorolfine.

Amorolfine is a structurally unique (see Fig. 3), topically active antifungal agent, which possesses both fungistatic and fungicidal activity *in vitro*. Its spectrum of *in vitro* activity includes dermatophyte, dimorphic, some dematiaceous and filamentous fungi, and some yeasts. In clinical trials, application of amorolfine 5% nail lacquer once or twice weekly for up to 6 months produced mycological and clinical cure in approximately 40–55% of patients with mild onychomycosis 3 months after cessation of therapy. Overall cure and improvement was observed in approximately 85–90% of patients with superficial dermatomycoses following treatment with amorolfine 0.25% cream for up to 6 weeks[44,45,46,47, 48,49,50].

Amorolfine blocks and depletes ergosterol and accumulation of ignosterol in fungal cytoplasmic membrane. Fungal cell wall becomes thicker and chitin deposits are formed inside and outside the cell wall[51]. It is believed that amorolfine exerts fungicidal activity on dermatophytes. It is also active against other filamentous fungi that cause onychomycosis, such as *Scytalidium* spp. and *Scopulariopsis* spp.[52,53].

## Azole Compounds

Azole compounds include imidazoles and triazoles. Basically, they are structurally and functionally similar. The introduction of the azoles (ketoconazole, itraconazole, and fluconazole) and the allylamine, terbinafine, led to improved cure rates and a broad spectrum of activity. Pharmacokinetic studies have shown that the newer oral agents penetrate the nail within approximately 1–2 weeks after the start of therapy and remain for several months after the end of treatment[54,55]. However, there are some patients who may exhibit poor response or failure to these newer drugs. The reasons for the nonresponsiveness are not fully understood. However, the possible explanations for this may be due to patient characteristics, the type of organisms causing or associated with the onychomycosis, individual nail characteristics, and/or local diseases involving the nail[56]. The azoles block ergosterol synthesis at one or more sites with the accumulation of 14-methyl sterol (which replaces ergosterol in the plasma membrane causing selective leakage and increased osmotic sensitivity). They also disrupt chitin synthesis. All effects are due to the binding to cytochrome P-450. Several derivatives of azole compound are available in the market. These include butoconazole (suppository, topical), clotrimazole (topical), econazole (topical), fluconazole (oral, topical), itraconazole (oral), ketoconazole (oral, topical), miconazole (IV, intrathecal, topical), oxiconazole (topical), sulconazole (topical), and terconazole (suppository, topical). Among these azole drugs, only four available oral antifungal agents (ketoconazole, itraconazole, terbinafine, and fluconazole) will be discussed as far as this paper is concerned.

However, it must be emphasized that based on literature search, only itraconazole, and fluconazole are highly recommended even for the most severe cases of onychomycosis[57,58,59]. Among these newer agents, oral itraconazole and oral terbinafine look promising to substantially increase cure rates, while shortening treatment duration. Oral terbinafine is potently fungicidal against dermatophytes and has proven efficacious with regimens as brief as 12 weeks, when the nail is not 100% involved[1]. The pharmacokinetic characteristics of both these drugs have made short treatment times effective. Patients with a culture-proven dermatophyte infection of the toenails, receiving terbinafine 250 mg daily for 12 weeks, have a 70% chance of cure[60]. The risk of relapse is probably less than 10%, and this may be prevented by a further course of terbinafine should the expected increasing length of unaffected nail growth stall or cultures become positive[60].

### Itraconazole (Sporanox)

Itraconazole is a triazole antifungal agent (see Fig. 4). As with other triazoles, it has five-membered ring structures containing three nitrogen atoms[61]. It is available in oral capsule form, oral solution in cyclodextrin, and intravenous formulation in hydroxy-propyl-beta-cyclodextrin. Itraconazole is a highly lipophilic compound. While it achieves high concentrations in fatty tissues and purulent exudates, its penetration into aqueous fluids remains very limited. Gastric acidity and food influence the absorption of

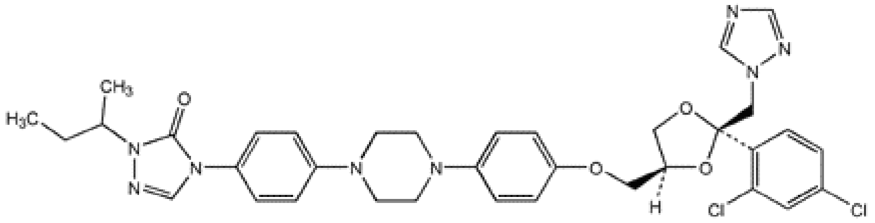

**FIGURE 4**. Chemical structure of itraconazole (Sporanox).

oral formulation[63]. Absorption of itraconazole oral capsule is variable and unpredictable. Oral solution of itraconazole is superior to the oral capsule in terms of absorption and bioavailability. The oral solution has 30% better bioavailability than the capsules and achieves higher serum concentrations[64,65,66, 67,68].

Haneke et al.[69] reported that clinical response rates and mycological cure rates at study end point were 89.0 and 68.4%, respectively, for toenails; 91.4 and 85.3%, respectively, for group A fingernails; and 84.4 and 77.1%, respectively, for group B fingernails. For itraconazole (pulse), the regimen is 200 mg twice daily for 1 week on, 3 weeks off, repeated for three consecutive pulses and with fluconazole the regimen is 150–300 mg once weekly given for a usual range of 6–12 months or until the nail plate has grown out. In some instances, if extra therapy is required, one suggestion is that 4 weeks of terbinafine or an extra pulse of itraconazole are given between months 6 and 9 from the start of therapy. Once cure has been achieved, it is important to counsel patients on the strategies of reducing recurrence of disease[3,70].

The therapeutic effects of intraconazole with intermittent pulse were judged by the following observations regularly and analyzed from the factors as follows: age, growing speed of nails, accompanied diseases, family history, trauma of nails, infection ways of the pathogens, manifestation of the injury, pathogens, and duration of the treatment[71]. The results showed that the recovery rate was higher in younger patients ($p < 0.01$) with a quicker recovery rate ($p < 0.001$) and a lower recurrent rate ($p < 0.01$), as well as in those with quicker growing speed of newborn nail. The patients with onychomycosis caused by *T. rubrum* had a higher recovery rate ($p < 0.01–0.001$) no matter whether the treatment duration was prolonged. The patients with diabetes mellitus or hyperhidrosis, as well as with positive family history or basic nail diseases such as trauma and paronychia, had a lower recovery rate and the curative effects were not satisfactory. The treatment duration is sometimes prolonged in order to increase the curative effects and decrease the recurrence under such conditions as following: old patients above 60 years; patients with low-growing-speed new-borne nails; patients with thumb and big toe injury and ingrown nail; patients with diabetes mellitus, hyperhidrosis, or Renauld's phenomenon; patients with nail trauma before or during the treatment; patients with onychomycosis caused by *Candida* or *Aspergillus*[71,72,73,74].

As with all azole antifungal agents, itraconazole works principally by inhibition of cytochrome P450 14a-demethylase (P45014DM). This enzyme is in the sterol biosynthesis pathway that leads from lanosterol to ergosterol[62]. Itraconazole is effective in the treatment of infections due to most yeasts and molds such as *Aspergillus* isolates and a subset of fluconazole-resistant *Candida* strains. However, the inconsistent bioavailability of the capsule limits the use of that form and the oral solution is preferred[75].

## Ketoconazole

Ketoconazole is effective in dermatophyte infections, but requires long-term therapy. Although it is a specific drug for blastomycosis, coccidioidomycosis, and candidiasis, it requires acidic conditions to dissolve and does not enter the central nervous system. Ketoconazole is no more effective than griseofulvin in toenail infections and there are significant problems with hepatotoxicity[38]. Ketoconazole has been the oral antifungal traditionally used for onychomycosis, but the agent is associated with relatively low cure rates (see Fig. 5). The adverse effects and drug interactions include:

- Nausea, vomiting
- Allergic rash
- Hormone imbalance, menstrual disturbances
- Fluid retention
- Hepatitis
- Teratogenic
- Inhibits drug metabolism
- Absorption reduced by $H_2$ antihistamines and omeprazole and antacids

Above all, ketoconazole is restricted in use by a rare incidence of hepatotoxicity[72,73,74]. While ketoconazole was more widely used before the development of newer, less toxic and more effective triazole compounds, fluconazole and itraconazole, its use has now been limited. It now appears as an alternative drug for specific indications.

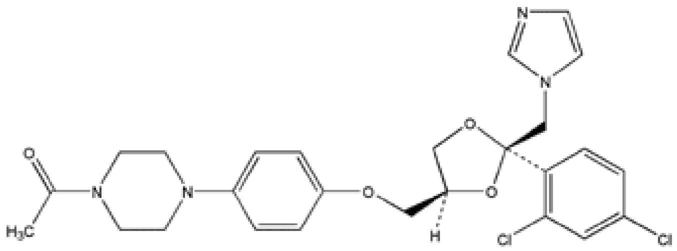

**FIGURE 5**. Chemical structure of ketocnazole.

## Fluconazole (Diflucan)

Few studies of fluconazole in nail infection have been carried out. These new agents appear to be safe and results thus far suggest that they will soon overtake griseofulvin as the drug of choice in the oral therapy of nail infection[76,77]. Fluconazole is a widely used bis-triazole antifungal agent. As with other triazoles, it has five-membered ring structures containing three nitrogen atoms (Fig. 6).

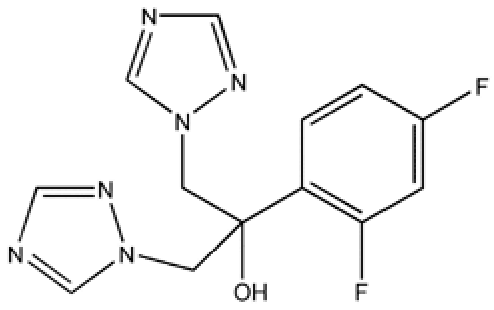

**FIGURE 6.** Chemical structure of fluconazole (Diflucan).

As with all azole antifungal agents, fluconazole works principally by inhibition of cytochrome P450 14a-demethylase (P45014DM). This enzyme is in the sterol biosynthesis pathway that leads from lanosterol to ergosterol[78,79,80]. Fluconazole is generally considered to be a fungistatic agent, principally active against some species of *Candida* and *Crytococcus*, blastomycosis, *Sporothrix schenckii*, and is sometimes used a second-line agent in these diseases. Fluconazole has no meaningful activity against *Aspergillus* spp. and most other molds or fungi[81,82].

## *Ciclopirox*

The chemical name for ciclopirox is 6-cyclohexyl-1-hydroxy-4-methyl-2 (1H)-pyridone with the empirical formula $C_{12}H_{17}NO_2$ and a molecular weight of 207.27 (Fig. 7). The mechanism of action of ciclopirox has been investigated using various *in vitro* and *in vivo* infection models and it was suggested that ciclopirox acts by chelation of polyvalent cations ($Fe^{3+}$ or $Al^{3+}$) resulting in the inhibition of the metal-dependent enzymes that are responsible for the degradation of peroxides within the fungal cell.

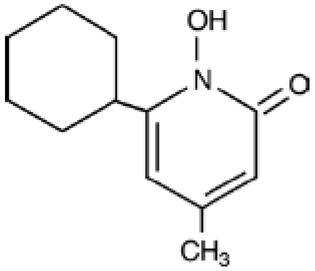

**FIGURE 7.** The chemical structure of ciclopirox.

Ciclopirox 8% nail lacquer is approved by the U.S. Food and Drug Administration (FDA) for the management of mild-to-moderate dermatophytic onychomycosis not involving the lunula[84].

Treatment with ciclopirox nail lacquer reduced the mean affected nail area from 64.3% at baseline to 41.2% at 3 months and 25.7% at 6 months. At 3 months, onychomycosis was rated as improved in 88.7% of patients, but unchanged in 9.8% and worse in 1.5%. The efficacy of ciclopirox nail lacquer was good in 62.0% of patients, satisfactory in 23.9%, and unsatisfactory in 14.1%, and the adverse events were mild to moderate with no serious events reported [83,84].

## Flucocytosine

Flucocytosine is a pyrimidine analog (Fig. 8). It is deaminated to fluracil where it is either (1) incorporated into RNA in place of uracil, where it inhibits protein synthesis or (2) metabolized to 5-fluorodeoxy-uridylic acid, where it inhibits thymidylate synthetase, thus blocking DNA synthesis.

Flucocytosine works by being taken up into the fungal cell by means of an enzyme called permease. It is then converted to 5-fluorouracil (5-FU) by cytosine deaminase and eventually 5-FU inhibits thymidylate synthase so that 5-FU triphosphate is synthesized and incorporated into RNA. Although it is against systemic fungi, mainly *Candida* and *Cryptococcus*, it is fungistatic and does well in combination therapy with amphotericin B (crytococcal meningitis) and with itraconazole (chromoblastomycosis). Unfortunately, it has some side effects namely:

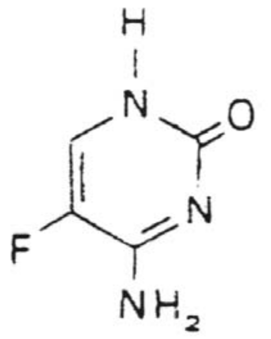

**FIGURE 8.** Chemical structure of flucocytosine.

- Nausea, vomiting, colitis
- Bone marrow suppression
- Thrombocytopenia
- Alopecia
- Decreased liver function

## Allylamines: Terbinafine (Lamisil)

Allylamines are fungicidal in activity against dermatophytes. They inhibit squalene-2, 3-epoxiase. The most important of the allylamines used in the treatment of onychomycosis is terbinafine. Terbinafine (Fig. 9) is an allylamine and a synthetic antifungal agent. It is highly lipophilic in nature and tends to accumulate in skin, nails, and fatty tissues[27,85,86]. The allylamine, terbinafine is particularly effective in the treatment of dermatophyte onychomycosis, with a much shorter treatment period than griseofulvin. Cure rates of well over 80% have been noted in fingernail and toenail infection during treatment periods of 6 and 12 weeks, respectively. Terbinafine, like itraconazole, diffuses more rapidly into the stratum corneum and nail and is more effective with short-term treatment[47]. Terbinafine is both a safe and effective first-line agent with reported overall cure rates of 50–90% for dermatophyte-related onychomycosis[72,73,74].

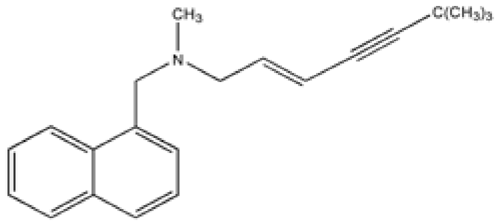

**FIGURE 9.** Terbinafine (Lamisil).

As with the other allylamines, terbinafine inhibits ergosterol biosynthesis via inhibition of squalene epoxidase. This enzyme is part of the fungal sterol synthesis pathway that creates the sterols needed for the fungal cell membrane. Terbinafine is mainly effective on a specific group of fungi, dermatophytes. Terbinafine yields lower MICs compared to fluconazole, itraconazole, and griseofulvin[86,87]. A total of 74% of patients treated with 12 or 24 weeks of terbinafine achieved a successful clinical outcome. Approximately 11% of terbinafine responders showed evidence of relapse 18–21 months after cessation of treatment. The clinical management of severe onychomycosis is known to be a long and difficult task[88]. Terbinafine has *in vitro* activity also against most *Candida* spp., *Aspergillus* spp., *Sporothrix schenckii*[87,89], *Penicillium marneffei*[90], *Malassezia furfur*[91], *Cryptococcus neoformans*[92], *Trichosporon* spp. and blastoschizomyces[93].

## Combination Therapy in Onychomycosis

Combination therapy with an oral antifungal agent may reduce the duration and cumulative dosage of oral therapy. Onychomycosis is difficult to eradicate; by using and combining different treatments (systemic itraconazole, systemic terbinafine, topical terbinafine after nail plate avulsion, and ciclopirox nail lacquer), it was possible to cure only 69.2% of patients with *Scopulariopsis brevicaulis* onychomycosis, 71.4% of patients with *Acremonium* onychomycosis, and 40% of patients with *Fusarium* onychomycosis. *Aspergillus* onychomycosis, on the other hand, responded very well to therapy and patients are usually cured after systemic or topical treatment[27,94,95]. The combination of a nail lacquer with an oral antifungal agent may further improve efficacy rates in certain clinical presentations (e.g., among those individuals with severe onychomycosis) while topical agents have a favorable adverse events profile[94]. For example, the management of onychomycosis using topical agents has improved with the introduction of ciclopirox and amorolfine nail lacquers[26,27,96]. Terbinafine, when combined with fluconazole, has occasionally been successful in treatment of oropharyngeal infections due to fluconazole-resistant *Candida* spp.[97]. The use of ciclopirox nail lacquer solution 8% as an adjunct to oral antifungal therapy may widen the spectrum of activity of the combination, because of the broad spectrum of coverage provided by the lacquer. The use of combination therapy may be synergistic in terms of efficacy, enabling a reduction in the duration and cumulative dosage of oral therapy. This could result in a decrease in the frequency and severity of systemic adverse effects associated with the oral antimycotics and the need to be vigilant about drug interactions[42,98,99,100,101,102,103].

However, there is good evidence to suggest that a combination of oral and topical therapies, when given at the same time, yield excellent clinical outcomes, although there remains a need for more effective topical agents with greater nail penetration and more effective oral antifungal agents[104,105,106,107,108]. Fluconazole, itraconazole, and terbinafin derivatives can safely be applied in combination with certain cauterants[109]. Combined therapy, such as the application of oral antimycotics and antifungal lacquers, or oral treatment accompanied by the surgical, laser, or chemical removal of the affected nail regions, can improve the results. Several types of combination can be considered, including the use of oral or topical drugs and the concomitant use of surgical techniques, all of which have a place in the treatment of onychomycosis. Synergy between antifungal drugs is well recognized[95,110,111,112]. However, combinations of antifungals are yet to be widely adopted to treat onychomycosis. The rationale for the combination of topical and oral therapy in the treatment of onychomycosis is that the systemic antifungal reaches the infection area via the nail bed and the topical agent is absorbed through the nail surface[85]. The combination of antifungals used should ideally be synergistic in their mode of action. Synergy between amorolfine and other antifungals has been demonstrated *in vitro*. This is presumably due to differences in the precise point of action of the drugs on the fungal cell membrane, where they inhibit the synthesis of ergosterol. Thus, combinations can result in increased antifungal activity at lower concentrations of both drugs. The potential exists therefore for combinations of antifungals to achieve higher cure rates in onychomycosis, in a shorter time than is currently possible[85,113,114].

## CONCLUSIONS

The drug treatments and persistence of onychomycosis have been evaluated. The overall literature findings showed that previously, the treatment of onychomycosis was discouraging, mainly due to a relatively low success rate, the need for prolonged therapy, and the laboratory monitoring necessary with the traditional oral antifungal agents like griseofulvin and ketoconazole.

Apparently, the advent of a new generation of oral antifungal drugs, including two triazoles (itraconazole and fluconazole) and an allylamine (terbinafine), has greatly improved the outlook for patients with fungal nail infections, particularly those with toenail involvement. Some newly approved onychomycosis drugs by the FDA seem to have broad-spectrum activity, for example, the azoles such as itraconazole. Numerous studies have demonstrated its efficacy when administered either continuously for 3 months or in “pulse” dosing. Research findings also suggest that fluconazole and terbinafine are promising, although their spectrum of activity is not as broad as that of itraconazole. An increased understanding of nail pharmacokinetics has led to the development of safer, more effective systemic therapies for onychomycosis, such as itraconazole, fluconazole, and terbinafine.

## BIOSKETCHES

**Andrew W. Campbell, MD** is the Chief Executive and Medical Director of the Medical Center for Immune and Toxic Disorders, Spring, TX. Hs is also a specialist in neuroimmunotoxicology.

**Ebere C. Anyanwu, PhD** is a research scientist in the Medical Center for Immune and Toxic Disorders and also Adjunct Professor of Anatomy and Physiology at the North Harris and Montgomery College District, Houston, TX. E-mail: ebereanyanwu@msn.com

**Mohammed Morad, MD** is a specialist in family medicine, lecturer in family medicine at the National Institute of Child Health and Human Development; Division of Community Health, Ben Gurion University of the Negev and the medical director of a large area clinic in the city of Beer-Sheva. Publications include those on Bedouin health, health aspects, spiritual health and aging in persons with intellectual disability, and he is a presenter on topics like health policy and services for disadvantaged at national and international conferences. E-mail: morad62@barak-online.net
